# Professor Litton’s Introductory

**Published:** 1844-02

**Authors:** 


### PROFESSOR LITTON'S INTRODUCTORY.

This is a glowing discourse by Professor Litton, pronounced before the Medical Class of the St. Louis University, at the beginning of the lectures in November last. Dr. Litton is a scholar and a chemist of high promise, and the zeal which he displays in his introductory lecture is a guaranty for the fulfilment of all the hopes of his friends. He pleads the cause of science like a true votary.

His mind and heart are full of its beauty and its value. He invokes the commercial spirit of St. Louis to rear up a chemical school in that growing metropolis, which shall vie with the renowned institutions of other lands. A pupil of the great chemist of Giessen, Dr. Litton is animated by all his master's devotion to chemical science. We hope his appeals to the munificence of his fellow-citizens may not be made in vain. Y.

